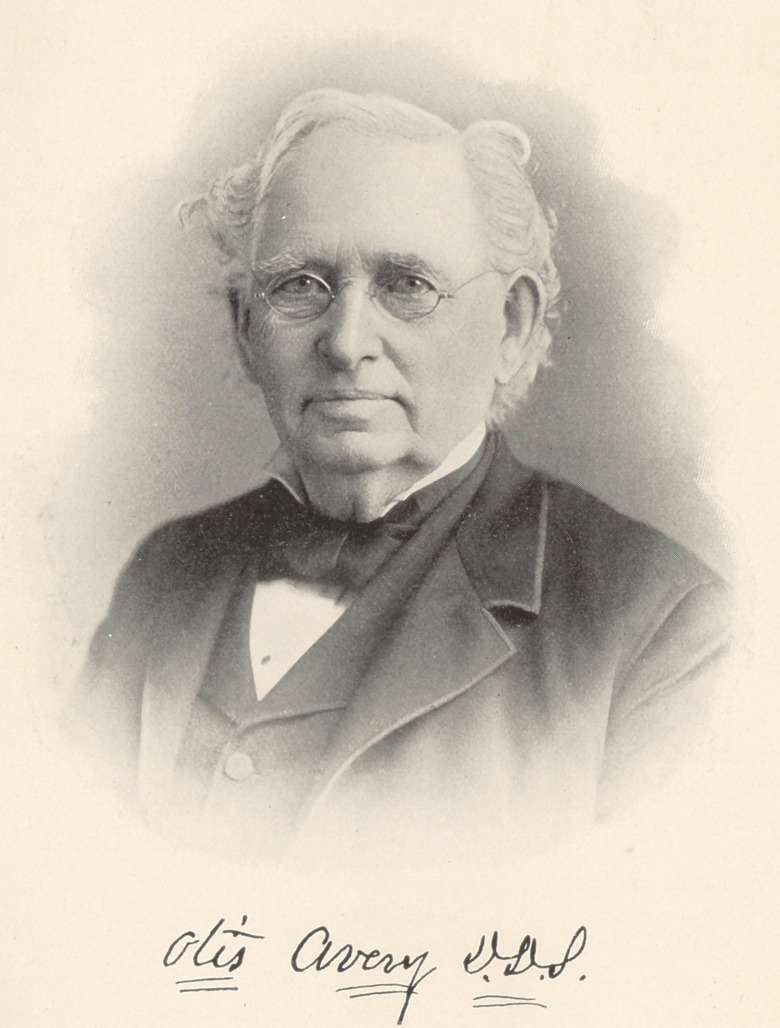# Reminiscences of Sixty-four Years of Practice

**Published:** 1897-07

**Authors:** Otis Avery

**Affiliations:** Honesdale, Pa.


Otis Avery D.D.S.

THE

### International Dental Journal.

Vol. XVIII. July, 1897. No. 7.

### Original Communications.[1]

### REMINISCENCES OF SIXTY-FOUR YEARS OF PRACTICE.[2]

BY OTIS AVERY, D.D.S., HONESDALE, PA.

Mr. Chairman and Gentlemen of the Convention,—I must beg the indulgence of this intelligent assemblage with regard to my remarks, inasmuch as they must, of necessity, be in a conversational manner of speaking, for the subject would not admit of anything oratorical, if I were capable of such, which I am not.

Having yielded to the wishes of a member of this Association to say something of the recollections of my earlier experiences in dentistry, allow me to say at the outset, that although I have had a busy life, it has been an uneventful one, and I never thought it worth while to keep any diary or memoranda of the occurrences. And, besides this, I am not a public speaker, and having been born in the first decade of this century, time has so impaired the vocal organs that it may be hard to understand what little I have to say.

In order to fully comprehend the character of the times, when dentistry had its advent, it would seem necessary to consider somewhat the condition of society and the trend of thought which moved the masses in the earlier years of this century, for we are living in a world altogether different from what it was then. I use

[1] The editor and publishers are not responsible for the views of authors of papers published in this department, nor for any claim to novelty, or otherwise, that may be made by them. No papers will be received for this department that have appeared in any other journal published in the country.

[2] Read before the Susquehanna Dental Association, at Carbondale, Pa., Wednesday, May 19, 1897.

the word *advent* advisedly with regard to dentistry, for, until the latter part of the last century and the first of this, the art was in its crudest form. The best they could do in supplying lost dentures was to fit a base plate and rivet to it teeth made from the tusk of the hippopotamus, neats teeth, or human teeth. Human teeth were most prized, notwithstanding they were procured by despoiling the dead. Even after the manufacture of porcelain teeth we were obliged to keep a stock of human teeth on hand to use as pivot-teeth for those who would have no other, because of their nearer match to natural ones.

Herodotus describes that the Egyptians confided the care of their teeth to a particular set of persons. But their operations must have been extremely limited and in no respect like that of the present day, for thousands of years may pass, and still the vast quantity of gold which already lies packed away in our cemeteries will assert itself.

At the risk of my remarks being considered rather irrelevant, I must state that in the earlier days of this century there was no mode of travelling upon land faster than a horse could get over the ground, and, of course, that regulated the speed by which intelligence was transmitted. Signals by semaphore were the only mode of telegraphy. Postage was regulated by the distance the letter was carried, and, in short, there was little of that intercommunication which exists at the present day,—little of the homogeneousness of society that we see now. There was, to a great extent, that exclusiveness which surrounds each little community, which is more or less marked in those who have had little communication with others.

The general awakening which took place at the beginning of this century must have had a cause. In dentistry the impulse could not have arisen solely from the mere fact that Billard[1], of France,

---

[1] Dr. William H. Trueman, at our request, writes in regard to this as follows: "Billard is a late writer. I do not recall his name as in any way connected with the introduction of porcelain teeth. Nicholas Dubois de Chemant is generally credited with making them a practical success. The Faculty of Medicine in the University of Paris gave him a certificate for his invention, March 5, 1789, and the Paris Royal Academy of Science, after carefully considering the rival claims, awarded him its approval June 10, 1789, shortly after which, I think, he received a patent in England.

"Porcelain teeth were commercially made in Paris, single teeth to be soldered on plates, as early, at least, as 1817. That year the elder Plantou brought some over to America."—[Ed.]

had discovered that teeth could be made of porcelain, and this had been improved upon by the Americans; and what more likely than that the utilizing the force which was known to exist in heated water and making it do the work of animal muscle should become the first step in the march of material progress. When Watt harnessed that power to a pump, demonstrating its utility, there was a general wish to make the power locomotive. And when the legislature of New York offered the exclusive right to navigate the Hudson River to the first person who would make a steam-driven boat that would run at the rate of three miles an hour, against the current, there was a general scramble for the prize. Fulton, having, with the aid of Chancellor Livingston, procured an engine built in France, brought it to New York, put it in a boat, and asked some of his friends to accompany him on his trial trip. Incredible as it may seem at this day, some were ashamed to be known to look with favor on such a chimerical scheme. And when some of the machinery got out of order, making it necessary to stop the boat to rectify it, they gave strong expression of their disgust and wished themselves on shore. They little thought they had witnessed the inauguration of a giant whose voice would wake the nations; who, with one foot on the sea and the other on the land, would revolutionize the commerce of the world; whose scream would be heard on hill-top and valley, amid the crags of the Rockies and the far-off plains of the Pacific. It is characteristic of the masses, to oppose or sneer at any step in advance of what may be called the common thought. It is not so marked at this day, but still this tendency exists. There is now, however, a growing disposition not to be surprised at anything. I well remember when it was not so; when there was a general scepticism of everything new.

I remember a conversation on the subject of stenography, some ten years before Isaac Pitman published his system of phonetics. A young man suggested that a better system of short-hand would be to base it upon sound instead of the old way of abbreviations, and arbitrary signs for phrases, and memory, and the people laughed at him. One, who had been a teacher for many years, said the thing was impracticable, for it would be physically impossible to take down a speech verbatim. Somewhere in the forties I was in New York, and saw a notice that there would be an exhibition of a new system of short-hand as taught by Andrews and Boyle. I attended and found a moderately sized hall partly filled with gentlemen, a large black-board at one end and a lad with a chalk crayon standing near it. The professor requested some one in the audience to read rather rapidly a paragraph from a newspaper. After the reading was finished the boy read what he had written, and, to the astonishment of the audience, it was verbatim as read from the newspaper. There it was; the impossible had been accomplished. And now the system is taken as a matter of course, for you find it in the busy marts of commerce, in the courts of justice, in religious assemblies, and public meetings, catching whatever falls from the speaker's lips on the point of a pencil and fastening it to paper.

So also it was with Daguerre. While struggling with the problem of how to fix the reflection of the sun's rays upon chloride of silver so as to form a picture, his friends became alarmed for his sanity, and called in experts to determine whether he was not following the phantom of a disordered brain. What is now the result? He has not only filled the world with beauty and gladness, but compelled the sun itself to deliver up secrets of being which could never have been known but for the camera.

Once more: Among my most vivid recollections is the change that was wrought by the construction of the Erie canal in the State of New York. There was a system of turnpikes from tide-water into the western portions of the newly settled country, a part of which was called the "Western Reserve,"—that portion of Ohio which Congress, just after the Revolutionary War, appropriated to the State of Connecticut to settle her claims on this part of Pennsylvania. These roads were teeming with emigrants going into that and adjacent States; and while living on one of these turnpikes I have heard the most blasphemous denunciations of the canal by those engaged in carrying produce and merchandise. The opposition ran so high that I believe, had the promoter (Governor Clinton) appeared among the people, he would have been mobbed for what was considered a waste of the public money upon a worthless ditch. But the canal was finished, and while it spoiled the turnpikes, it created an empire. And the sons and grandsons of these very men have, by popular vote, just decreed that millions more shall be expended on what their fathers termed "Clinton's worthless ditch."

I have cited these facts to try and throw a side-light upon those early times. In speaking of what I remember of the earlier days of dentistry I cannot, as I see, do better than give a narrative of my own experience, unpleasant as it is to speak in the first person and at the risk of being charged with egotism.

In the year 1833 I received my certificate of qualification to practise dentistry from my preceptor, Dr. D. C. Ambler, of Barclay Street, New York. You will bear in mind that at that time there was not a dental school in all the world, nor any institution where dentistry was taught. Neither was there a journal devoted to the art (for it was called an art in that day), and the only mode available for improvement was by comparing notes among several dentists, which was a kind of close corporation, keeping our several views within the charmed circle.

In that day the anæsthetic properties of sulphuric ether or chloroform were not known, and we were equally ignorant of all obtundents to relieve the pain of sensitive dentine. Our only recourse was to a keen excavator and keeping the cavity dry, which was sometimes very difficult to do, for we knew nothing of the rubber dam. Whenever we had a very severe case of sensitiveness we would fill the cavity with a pellet of cotton saturated with creosote and morphine, and dismiss the patient until the next day; but often when a patient submitted to a dental operation he would think he had fallen into the hands of the tormentors.

With regard to dental literature, you will often find now more practical information in one number of a leading dental journal of this day than all the dental literature of those days in the whole English language.

As it was at first supposed that each dentist would be obliged to make the teeth he used, it was a part of our education to manufacture them; and the apparatus for such purpose was no small part of the outlay in getting ready to practise. But four or five years cured us of that fallacy, as there is nothing in common between the high art of manufacturing porcelain teeth and the practice of our profession, or the skill in setting them.

While the demand produced the supply in a few years, at first we were obliged to make our own instruments, especially pluggers and excavators. Having furnished myself with what was then considered an ample outfit, composed, say, of a dozen of agate-handled pluggers and some four dozen mother-of-pearl handles with sockets for excavator-blades, all ferruled with gold and arranged in trays, they made a beautiful display; and having made them myself I felt justly proud of them. I took them to the town where I had lived some three or four years, and confidently expected to receive as liberal a support as a dentist as I had as a watchmaker and silversmith. But nobody seemed to be in want of my services in that line. I was a member of a religious denomination, and I asked a member, with whom I was on intimate terms, to call and look at my instruments. After contemplating them a little time, I found he did not enthuse any and wondered what was the trouble. At last, with a sigh, he turned and said, "Brother Avery, do not you think you would be doing the public and yourself more good by throwing these things into the river and going back to your old trade?"

To say that I was astonished is putting it mildly. I saw at once that he took a view of the matter which I had never thought of; that there was a moral principle involved in the occupation of dentistry. The church then discountenanced personal adornment much more than it does at this day, and held that anything which tended to foster the pride of life was to be shunned, as from the evil one. I soon found that the matter had been discussed and settled, among those good men, that I was going straight to perdition, and that they must make an effort to prevent such a catastrophe; for another member called to argue the question, and among other things he said that practising dentistry was going against and in the face of Providence. And also that it was not a respectable calling. I cited to him the fact that the best of men did not hesitate, when their eyesight failed, to supplement it with glasses, nor was it considered going against Providence for a man who had lost a limb to have an artificial substitute. And as for the respectability of the calling, I would try and make it so, so far as I was concerned, with much more in the same line. But anything I could say went for nothing. Our church in the town was but a charge belonging to a circuit, with no resident minister; the preacher visiting us only once a week. But he, being an intelligent man, soon put a stop to any further action against me; otherwise I do not know but they would have considered me a subject for discipline. Yet they were good men, and I thought, "let the righteous smite me," etc. It stirred up, however, an opposition to me such as they little expected. I had been somewhat active in our social meetings, and there was a class that looked with delight upon what they considered my fall and repudiation by the church. They "always knew I was a scoundrel," and "were not surprised that I should show it when it was my interest to do so." All of which I let pass; but when they ventured to charge me with crime, rather than be called to answer before a court, they were willing to acknowledge the charge malicious and false.

Finding I should get nothing to do in that neighborhood, with such a feeling against me, I determined—was compelled—to find some one who knew what dentistry was, and I started on my travels. I went from town to town by such conveyance as offered or could be procured. On my arrival at a place I would send a boy out with my card, stating that I could be consulted and would be happy to attend upon any who should need my services as a dentist, either at the hotel or at their residences. Then came an anxious waiting. I would display my instruments so they could be seen, and sometimes there would be half a dozen young fellows in to stare at me and gaze at the instruments. I never was very self-asserting, and that fact may have had something to do with it. But they seldom spoke to me, and I had too much self-respect to make a personal application for their patronage; though I maintained a sociable and friendly manner. Thus it was, day after day, in different towns and hamlets until I became heart-sick.

It seemed as though there was a conspiracy against me, for nobody wanted my services or seemed to know what dentistry was; they seemed to think my instruments were simply to look at. But at one place where I stopped, when they were looking at and talking about them (they had found that I had made them), one of the company said, "I do not know what them tools are for, but this I do know, that a man who could make them knows how to use them." He was the first to grasp my motive in displaying them. With half a mind to give up the struggle and turn back, I felt as a man must feel who had expended all that he had and could borrow on a cargo of goods which nobody wanted, or would take as a gift. I was convinced of the fact that some had looked at the matter as a show; as, when I stopped at one place, which was nothing more than a hamlet, I found a crowd with an air of expectancy standing around, and as I stepped into the tavern the landlord said, "Your things have not come yet." It appeared that some wag had told them there was a show coming and to look out for it. As soon as we could feed the horse we drove to the next town, leaving them in expectation of a harlequin with his Punch and Judy. At another time I had employed a man to take me from a town, and after we had started he said to me, in a confidential tone, "How's business?" "Not much," I answered. "Oh, now," he replied, "you needn't say that, for you folks always pretend you have made nothing. I know one fellow who made a big swag about six months ago." "How did he make it?" I asked. "Why with his roulette," said he. I replied, "I am not in that business." "What business are you in?" he asked. "Dentistry," said I. He gave a snort of disgust, as much as to say, "I thought you were a gambler, but you are nothing but a dentist."

I had started out full of hope and expectation, but here I was with my money nearly all spent, and from an optimist I had become an inveterate pessimist, disgusted with myself and my surroundings. In this state I arrived at a town where I had been known when a boy, though I had never lived there, and sent out my cards, and was debating whether I should give up the struggle and take the next stage for home when there came a message from a wealthy Irish family in the neighborhood, requesting me to call at their house with my instruments. I found they were well versed in dentistry of the old style, but when the lady found that the base plate was made of gold, with porcelain teeth soldered on it, instead of an ivory plate with the teeth riveted to it, she immediately ordered an upper set. The younger branch of the family consisted of a son and his wife. Their teeth had been kept well filled. The wife, I found, was the prime mover in calling me to visit them. She insisted on her husband having his teeth cleaned, and tried in every way to find something for me to do so as to make a bill. Now it has always been a query with me whether it was not sheer compassion which induced her to have me call at their house; for, by some means, they had heard of my being in other places and how I had been received. At any rate, I have always since liked the Irish and kept for them a warm place in my heart, for they were the first to offer me any encouragement and kindly advice. I had hardly taken an impression before it was noised about town that Mrs. Penderghast was going to have a false set of teeth put in her mouth. While I was much elated, I tried to maintain my equanimity as though the ordering of a fifty dollar set of teeth was an every-day occurrence. Yet the sky looked brighter, the birds sang sweeter, and my feet took a more elastic spring. Hope returned and I decided to go on to Utica, where there was only a dentist and a half, for one of them divided his time between shaving his customers and extracting their teeth, with phlebotomy incidentally added. I found whom I intended to call on, and, fortunately for me, they were in want of my services, and they advised their friends to employ me for what dentistry they needed, so that I was amply paid for my visit there.

They urged me to open an office in the city, and I went so far as to look for suitable rooms, to be ready, if, on consulting my wife, we should conclude to take them. She had been kept advised of the condition of things, which had resulted in no money till I got to Utica, except the little produced by extracting a few teeth, and that generally caused an altercation, because I charged a shilling, whereas the doctors would charge only sixpence for extracting. She said my mistake was in going north instead of south; that if I had gone among the *bandits* of the Beech Woods I must certainly have succeeded. The term she used needs some explanation. Soon after we had moved to New Berlin we were at a social evening party, where a lady told of a visit of a friend who had travelled all the way from Philadelphia with their own conveyance, and their route necessitated their travelling through the Beech Woods of Pennsylvania, which was filled with all sorts of wild beasts, such as wolves, bears, and other ferocious animals, with men more savage and dangerous still, who made those woods their haunts. They were in constant fear for their lives, as they often saw men with guns and axes prowling around in the bushes; that they actually saw where one murder had been committed, marked by a pile of stones by the side of the road, with a lot of other trash of the same kind. After she had finished, the company sat spell-bound with horror and almost breathless to think there was such an awful place in this country. After a little my wife said, "Well, I was born and brought up in the Beech Woods and educated in the Beech Woods Academy, at Bethany, and never in all my life heard such stuff as that about my native county." The effect was electrical. If a native of the Feejee Islands had dropped down among them they would hardly have been more surprised, for here was a denizen of that terrible place sitting in their midst clothed and in her right mind. The lady, however, was not disposed to give it all up, so, turning to me, said there certainly was a murder committed, as she saw the pile of stones which marked the place. "Yes," I replied, "but both the murderer and his victim belonged in the State of New York, and all that the people of the Beech Woods had to do in the matter was to hang the murderer in accordance with the law."

Coming back to Wayne County as a dentist to my friends in Bethany (which still was the county seat), who knew me as having been the first to establish a watch-making and silversmith-shop in the county, they received me cordially, showing no intimation that they thought I had done anything reprehensible by becoming a dentist. Thus was my itinerary established, reaching from this point north to the Mohawk Valley, more than a hundred miles in length, and taking in most of the towns within twenty miles on each side. Except at Binghamton there was not, in all that region, a resident dentist. I soon found it necessary to drive my own conveyance to economize time, for as soon as I had finished the operations required and taken such impression as offered in one town, I started for the next, whether it was in the daytime or at night. In fact most of my travelling was at night. I would send out my cards, wait one day, and if nothing offered, after supper would start for the next place. Whenever the amount of business in a town fell to less than five dollars a day I started on my travels. By such means those wanting anything done were sure to be on hand. Sometimes there would be a general scramble for chances. All the mechanical work was done at home, so this and the manufacture of teeth took up all my time.

In that day there were men who had been eminent preachers in their prime, but were stranded,—silenced as much as if by decree of Conference or Synod because of their inability to pronounce many words of our language, made up as it is so largely of dental sounds. When they began to preach again, to their own delight and the edification of the public, my conscientious brethren admitted that dentistry was not altogether bad; that false teeth might be a help in speaking, but that was all; they were only good for that, but of no service in eating. I had put in a set of teeth for a physician of more than local repute, who, when bantered by a lady for being so proud as to have artificial teeth, which could be of no use to eat with, said to her, "Put your finger in my mouth and see whether they are all for looks." She did not want to repeat the experiment, certainly not until her finger healed. These things seem very trivial now, but at that time it was as much of a curiosity as the telephone was on its first introduction.

Thus we plodded on, discussing various problems, "in pursuit of knowledge under difficulties," when now and then there would start up, for example, a genius who knew it all and would deliver himself of such flashes of wisdom as this: "That as the decayed part of the tooth was always softer than the other portion, there was no sense in making so much ado about the temper of the excavators. It was only necessary to take a piece of steel wire, bend it to the shape wanted, then file it to an edge, and there you are."

Gentlemen of the Association, you are to be congratulated on being in the midst of a movement and growth of the profession which is truly phenomenal, and which is far-reaching in its beneficence, not only adding hundreds of years to the sum of human life, but making that life enjoyable through the practice of your profession, a profession which has already arrived at such a stage that the old name does not now express its full significance and scope. Who, then, dare guess what another three-quarters of a century will bring forth, as others shall take up the science as it will be when you lay it down. Considering what has already been accomplished we may reverently adopt the saying, "What hath God wrought?"

One word to the younger members of this Association with regard to what I have learned to be the best attitude to assume in relation to the practice of our profession. For a higher system of ethics you must look elsewhere. Be temperate in all things. You will find its advantage in steadiness of the nerves. Do not worry, for there is a higher power than you who will make all things even. Eliminate from your dental vocabulary the word *can't*, for you do not know what you can do until you try. When you go upon a vacation (for, if you have had anything like a full practice and have used your brains with your work, you will need rest), leave the shop behind, for if you take it with you it would be just as well to stay at home. Be honest both to yourself and to your patient. Humor their idiosyncrasies, as far as possible; you will find it the best policy. In all your work, whether at the chair or in the laboratory, bear in mind the sentiment involved in the answer of the heathen sculptor, who, when asked why he devoted so much skill and labor in finishing the back part of the statues he was preparing for a Grecian temple, as when placed in their niches nobody could see that side, replied, "The gods see them on all sides."

## Figures and Tables

**Figure f1:**